# tRNA Modifications: Impact on Structure and Thermal Adaptation

**DOI:** 10.3390/biom7020035

**Published:** 2017-04-04

**Authors:** Christian Lorenz, Christina E. Lünse, Mario Mörl

**Affiliations:** Institute of Biochemistry, Leipzig University, Brüderstraße 34, 04103 Leipzig, Germany; christian.lorenz@uni-leipzig.de (C.L.); christina.luense@uni-leipzig.de (C.E.L.)

**Keywords:** post-transcriptional modifications, pseudouridine, dihydrouridine, dimethylguanosine, methyladenosine, archaeosine, lysidine, methylguanosine, tRNA, tRNA structure

## Abstract

Transfer RNAs (tRNAs) are central players in translation, functioning as adapter molecules between the informational level of nucleic acids and the functional level of proteins. They show a highly conserved secondary and tertiary structure and the highest density of post-transcriptional modifications among all RNAs. These modifications concentrate in two hotspots—the anticodon loop and the tRNA core region, where the D- and T-loop interact with each other, stabilizing the overall structure of the molecule. These modifications can cause large rearrangements as well as local fine-tuning in the 3D structure of a tRNA. The highly conserved tRNA shape is crucial for the interaction with a variety of proteins and other RNA molecules, but also needs a certain flexibility for a correct interplay. In this context, it was shown that tRNA modifications are important for temperature adaptation in thermophilic as well as psychrophilic organisms, as they modulate rigidity and flexibility of the transcripts, respectively. Here, we give an overview on the impact of modifications on tRNA structure and their importance in thermal adaptation.

## 1. Introduction

The life of a transfer RNA (tRNA) molecule starts with a series of important maturation steps that can vary in their sequential order from case to case. Leader and trailer sequences are removed by a set of endo- and exonucleases, and in several tRNA precursors, splicing reactions excise intronic sequences [1,2,3]. Furthermore, in many organisms the sequence CCA, that represents the site of aminoacylation, is not encoded, but has to be added post-transcriptionally by CCA-adding enzymes [4]. While all primary tRNA transcripts are composed of the four standard RNA bases A, C, G and U, many of these nucleotides are modified, altering their properties in very different ways [5]. Currently, 93 post-transcriptional modifications are known, and the variety of their functions is at least similarly diverse and not fully understood. The complexity of such modifications ranges from simple methylations at the bases or the ribose to rather complex and large base hypermodifications, whose synthesis often requires a whole cascade of enzymatic reactions. Modifications can alter a tRNA’s shape in subtle ways, but can also lead to massive structural rearrangements. In addition, they ensure efficient translation by maintaining the anticodon loop structure and promoting correct codon-anticodon interactions, especially at wobble positions.

**Figure 1:**
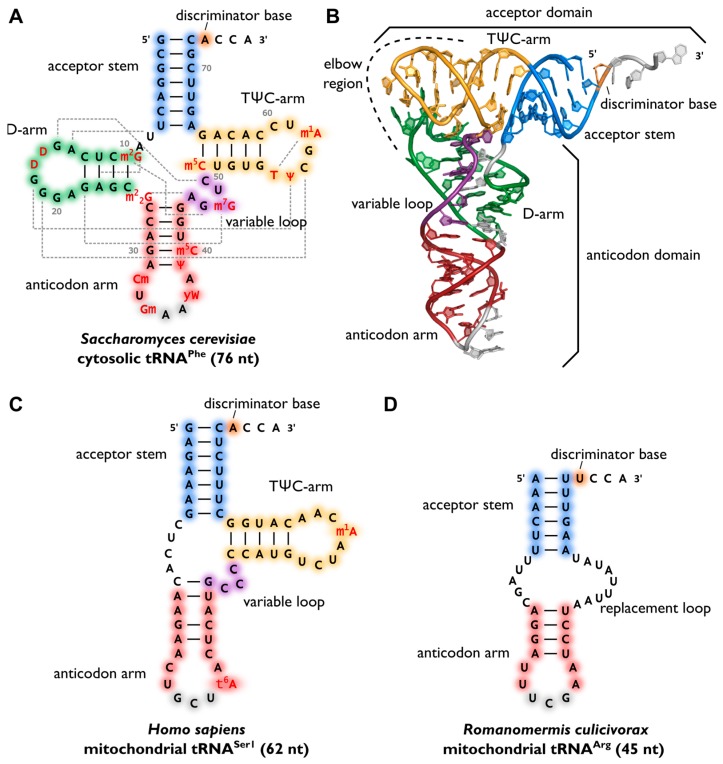
Variability of transfer RNA (tRNA) structures. (**A**) The canonical cloverleaf secondary structure of cytosolic tRNA^Phe^ from *S. cerevisiae* is shown with acceptor stem (blue), D-arm (green), anticodon arm (red), variable loop (purple) and TΨC-arm (yellow). The anticodon is labeled in grey, the discriminator base in orange and post-transcriptional modifications in red. Grey dashed lines indicate tertiary interactions based on structural data [11]. Base numbering corresponds to Sprinzl et al. [25] and length of the RNA is indicated in parenthesis; (**B**) The L-shaped tertiary structure of the cytosolic tRNA^Phe^ from *S. cerevisiae*. Protein Data Bank entry (PDB): 1EHZ [11]. The acceptor domain is composed of stacked T-arm and acceptor stem, whereas D- and anticodon arm form the anticodon domain. The region where both domains come together and interact with each other via tertiary base pairing is also called elbow region; (**C**) Secondary structure of human mitochondrial tRNA^Ser1^, which lacks the whole D-arm [26]; (**D**) Secondary structure of the mitochondrial tRNA^Arg^ from the nematode *Romanomermis culicivorax*, which lacks both D- and T-arm. Instead, we find a so-called replacement loop. It represents the shortest tRNA found in vivo [21].

After maturation, tRNAs have multiple interaction partners in their life cycle, ranging from aminoacyl-tRNA-synthetases to translation factors, ribosomes and mRNAs. Apart from synthetases, these interaction partners do not specifically act on one individual tRNA transcript or isoacceptor, but on all tRNAs, like the above mentioned CCA-adding enzyme [4]. Despite a high sequence variation, a cell’s tRNAs show a well-conserved cloverleaf-like secondary structure that was originally discovered in 1965 [6,7]. The cloverleaf consists of five parts (Figure 1A): the acceptor stem, containing the tRNA’s 5′- and 3′-ends, the D-arm, the anticodon arm, the variable loop and the TΨC-arm (T-arm). At the 3′-terminus, the tRNA carries the CCA-sequence, required for aminoacylation, tRNA positioning in the ribosome and translation termination [8,9,10]. In a conserved network of tertiary interactions, mostly between D- and T-loop, tRNAs fold into an L-shaped three-dimensional structure [11,12,13], which was first solved by Kim et al. in 1974 (Figure 1B) [14]. Furthermore, the tRNA elbow region, which is a remarkable part of the tertiary structure, has a hydrophobic character due to the surface-exposed base pair G19-C56. This special feature was shown to be important for the interaction with other RNAs and proteins, like RNase P, T-box RNAs or some aminoacyl-tRNA synthetases [15]. Anticodon and the amino acid-accepting CCA-end take the longest possible distance from each other. This conserved structure of a tRNA is essential for its recognition by other RNAs and proteins and, consequently, for its functionality. For example, the CCA-adding enzyme uses the acceptor domain for substrate recognition, whereas aminoacyl-tRNA-synthetases use several recognition elements like anticodon, acceptor stem or the discriminator position. This nucleotide is located immediately upstream of the CCA triplet and is not involved in base-pairing (except in tRNA^His^, where it pairs with an extra nucleotide at the 5′-end). While interacting with other molecules, like T-box elements or aminoacyl-tRNA synthetases, the tRNA slightly adapts its structure, reflecting the need for a certain flexibility (reviewed in [7,16]).

Surprisingly, not all tRNAs fold into the canonical cloverleaf structure. Especially many mitochondrial tRNAs are reduced in length and sometimes completely lack the D- or T-arm (Figure 1C). In mitochondria of nematodes, this situation is carried to an extreme, as tRNAs lacking one or even both arms seem to be the rule (Figure 1D) [17,18]. As a result, the tertiary interaction network between D- and T-loop cannot form or is strongly reduced, as it was shown for tRNA^Phe^ from *Bos taurus* [19]. Similar results were obtained for human mitochondrial (mt)-tRNA^Asp^ [20]. Yet, these bizarre tRNAs are efficiently and correctly processed by the CCA-adding enzyme in vitro as well as in vivo [21,22]. Studies on engineered tRNA^Asp^ from yeast, mimicking a mt-tRNA by lacking both D- and T-arm, indicate that such a truncated tRNA can be aminoacylated [23]. As the tertiary interactions between the bases of D- and T-loop seem to be essential for a stable fold, it is still not clear how these dramatically reduced tRNAs adapt their functional 3D structure. However, there is experimental evidence that several post-transcriptional base modifications play an important role in the formation of a functional L-shaped tertiary structure of such bizarre tRNAs [24].

Among all types of RNA, tRNAs show the highest density of modifications, rendering them excellent models for the investigation of these nucleotide alterations. The database tRNAmodviz offers interactive maps that show the distribution of modifications in tRNAs [27]. The highest level of post-transcriptional tRNA modifications is found in Viridiplantae (up to 23.7%), while cytosolic tRNAs of single-celled eukaryotes and Gram-positive bacteria carry bases that are modified up to 16.6% and 6.6%, respectively [27]. Here, one has to be aware that databases only report whether a modification has been generally observed at a certain position, but not the actual degree of modification. For example, position 54 in all tRNAs is usually completely methylated (100%) to ribothymidin (abbreviated m^5^U or T), which incidentally also gave rise to the “TΨC”-arm nomenclature. In thermophiles however, the additional C2 thiolation in the pyrimidine ring (m^5^s^2^U or s^2^T) can vary from 0 to 100%, depending on the growth temperature [28,29].

**Figure 2:**
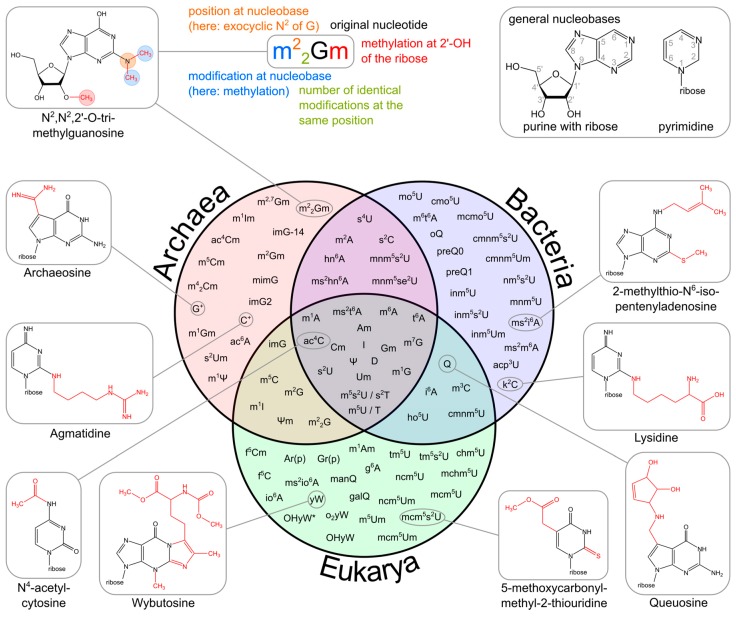
Variability of tRNA modifications. The upper part of the image illustrates the systematic abbreviation of RNA modifications with *N*^2^,*N*^2^,2′-*O*-trimethylguanosine (m^2^_2_Gm) as an example and also shows the atom numbering in the purine and pyrimidine rings as well as in the ribose. An abbreviation in front of the base letter describes a base modification, whereas letters after the base stand for ribose alterations. Superscripted numbers specify the position at the base and subscripted numbers indicate the frequency of identical modification at the same position. Abbreviations are as follows: ac—acetyl, acp—aminocarboxypropyl, chm—carboxyhydroxymethyl, cmo—oxyacetic acid, cmnm—carboxymethylaminomethyl, f—formyl, g—glycinyl, gal—galactosyl, hn—hydroxynorvalylcarbamoyl, ho—hydroxy, i—isopentenyl, inm—isopentenylaminomethyl, io—*cis*-hydroxyisopentenyl, m—methyl, man—mannosyl, mchm—carboxyhydroxymethyl methyl ester, mcm—methoxycarbonylmethyl, mcmo—oxyacetic acid methyl ester, mnm—methylaminomethyl, mo—methoxy, ncm—carbamoylmethyl, nm—aminomethyl, r(p) —5-*O*-phosphono-b-d-ribofuranosyl, s—thio, se—seleno, t—threonylcarbamoyl, tm—taurinomethyl. The Venn diagram summarizes data collected from the RNA modification database and contains the 93 post-transcriptional modifications that are found in tRNAs [5]. Some examples mentioned throughout the text are shown with their chemical structure.

Of the 112 modifications found across all types of RNAs, 93 different modifications are present in tRNAs [5]. However, the tRNA modifications differ among the three domains of life in terms of density as well as composition. In general, eukaryotic tRNAs are more heavily modified than their bacterial homologs, while parasitic and plastid tRNAs show the lowest density of modifications [27,30]. Simple modifications like pseudouridine (Ψ) or 1-methyl adenosine (m^1^A) are found in all domains. Most hypermodifications however, are specific to one domain and represent markers of evolution [5,7]. For example, the 7-deaza-guanosine derivative archaeosine is only found at position 15 in archaeal tRNAs, but not in that of other domains [31]. Generally, more than half of the modifications found in tRNAs are domain specific, only one fifth is spread across all domains and another fifth is found in the overlapping regions of two domains (Figure 2). Doubly modified nucleosides of the type xNm, like s^2^Um or m^2^_2_Gm, are only found in archaea. These nucleosides combine a 2′-*O*-methylation of the ribose with a chemical alteration of the base (Figure 2). Several of the underlying simple modifications are found in the overlapping regions between the individual domains of life (Venn diagram Figure 2). Intriguingly, some hypermodifications are isoacceptor specific, like wybutosine (yW, see Figure 2 for chemical structure) which occurs only at position 37 in tRNA^Phe^, where it stabilizes codon-anticodon interactions [32,33]. As the synthesis of hypermodifications follows a complex metabolic pathway, their intermediates are usually not independent modifications, as shown for nm^5^U, cmnm^5^U and mnm^5^U or the queuosine (Q) and wybutosine (yW) precursors preQ0, preQ1, oQ, gluQ, imG, imG2, mimG, OHyW, and OHyW*.

The effect of modifications on the structural stabilization of the anticodon loop, the decoding of the wobble position and prevention of frameshifting in translation has been investigated extensively and reviewed elsewhere [34,35,36,37]. The variety of modifications [38] and their impact on the tRNA structure has also been greatly reviewed [39,40]. Here, we want to summarize these data and focus on the adaptation to environmental factors, especially temperature.

## 2. Structural Impact of Modifications

**Figure 3:**
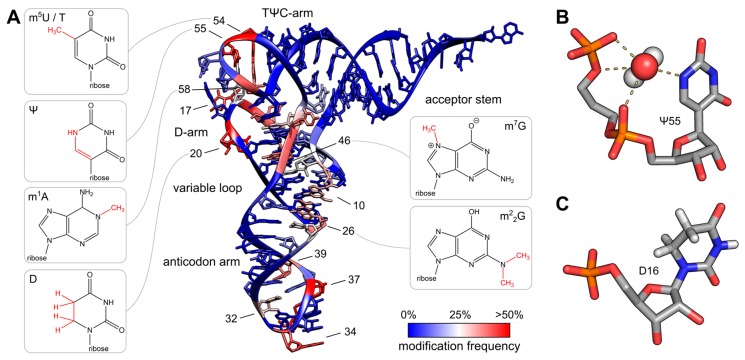
Post-transcriptional modifications in tRNA. (**A**) The colored tRNA structure shows the modification frequency of each base. The modification data were taken from the tRNAmodviz database [27] and plotted on the crystal structure of tRNA^Phe^ from *S. cerevisiae* [11]. Blue colored bases are rarely modified; red colored bases are modification hotspots. tRNAs possess two regions with high modification levels—the anticodon loop (especially positions 34 and 37) and the core or elbow region, where D- and T-loop bases interact with each other and stabilize the tertiary fold. For some important positions, the chemical structure of the most frequent modification at this position is shown; (**B**) Three dimensional structure of pseudouridine at position 55 of tRNA^Phe^ from *S. cerevisiae*. The additional H-bond donor at N1 interacts with the 5′-adjacent phosphates via a coordinated water molecule. The hydrogen bound to N1 was not resolved in the crystal structure. The ribose shows a stabilizing C3′-endo conformation. PDB: 1EHZ [11]; (**C**) Three dimensional structure of D16 in the D-arm of tRNA_i_^Met^ from *Schizosaccharomyces pombe*. The C5-C6 bond of dihydrouridine is reduced, which leads to a non-planar structure of the base. The ribose takes the less stable C2′ -endo conformation. PDB: 2MN0 [41].

The variety of post-transcriptional modifications can be classified into two groups according to their complexity. The first group comprises the majority of modified bases, which have simple methylations at the ribose or base moiety that are usually introduced by a single enzymatic reaction. Simple modifications can be found at almost every position of the tRNA molecule with a high density in the tRNA core region, where tertiary interactions between D- and T-arm stabilize the three-dimensional fold (Figure 3A). The second group includes complex modifications, whose synthesis requires the sequential activity of several enzymes. These more complicated alterations, termed hypermodifications, are mainly found in the anticodon loop, where they preserve its structure as a prerequisite for efficient translation (Figure 3A).

The impact of modifications on a tRNA’s structure can be investigated by comparative studies of the native, modified tRNAs and in vitro synthesized non-modified tRNAs obtained by T7 RNA polymerase-driven transcription [42]. Interestingly, most in vitro transcripts are functional in aminoacylation (references in [43]) or CCA-incorporation assays [44,45,46]. This highlights that most modifications are not strictly required for a functional tRNA and that unmodified tRNAs can also adopt a biologically active structure. Yet, one has to keep in mind that modifications are not the only factor that has an impact on the 3D structure of an RNA. Especially bivalent metal ions such as Mg^2+^ are equally important and can promote a native fold in unmodified run-off transcripts or increase the melting temperature of native tRNAs [47,48,49]. Hence, the conditions for in vitro structure analyses have to be selected carefully.

Based on the impact on the tRNA structure, the effects of modifications range from the reorganization of base pairs, or even complete structural domains to equally important fine-tuning of restricted local elements, like base stacking or loop flexibility.

### 2.1. Global Structural Effects (Domain Rearrangements)

Put simply, RNA is composed of a small number of individual building blocks (nucleotides A, C, G and U), allowing the formation of many different base pairs that can lead to alternative secondary and tertiary structures. As a consequence, an RNA will not always fold into its biologically active conformation if other, alternative structures show a similar level of free energy. This “RNA folding problem” [50] even worsens if the RNAs show a pronounced nucleotide bias as observed for thermophilic organisms (GC-rich) and some mitochondrial genomes (AT-rich).

**Figure 4:**
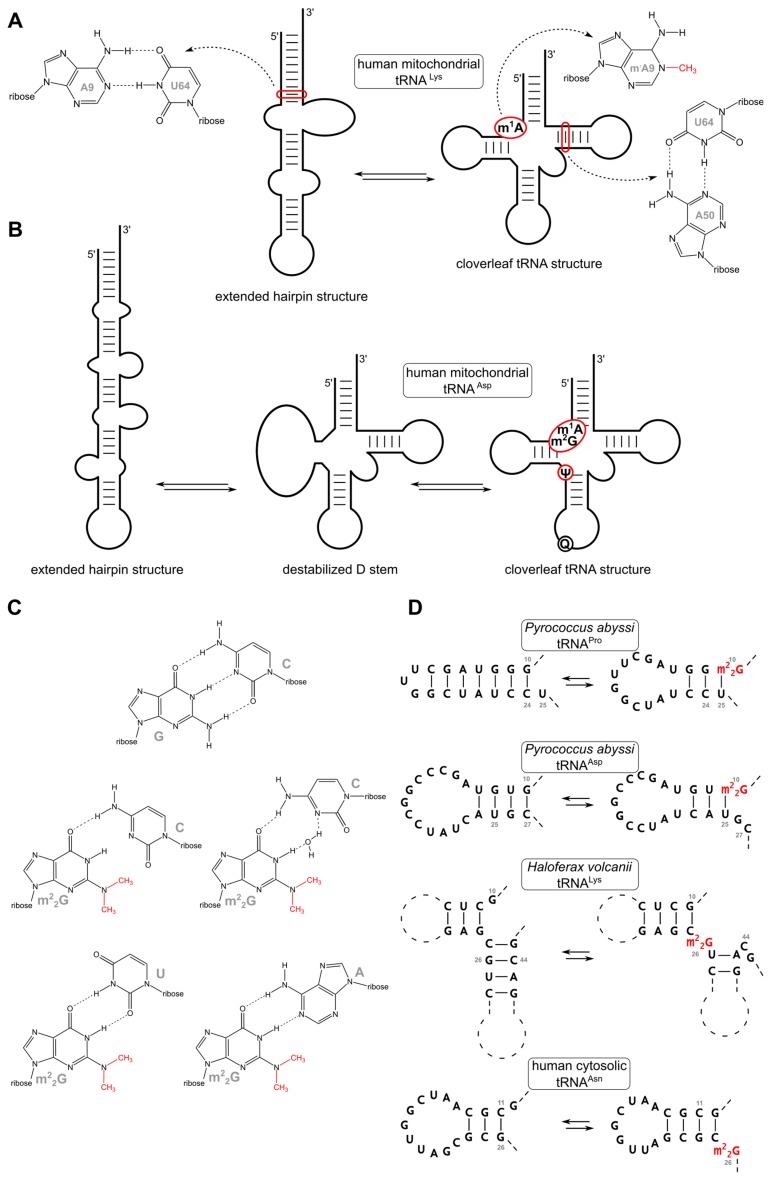
Modifications direct a tRNA secondary structure. (**A**) The in vitro transcript of human tRNA^Lys^ shows an extended hairpin structure due to missing modifications. The introduction of N^1^-methyladenosine (m^1^A) at position 9 interrupts a base pair with U64 and forces the canonical folding of the tRNA. U64 now pairs with A50 [51,52]; (**B**) The unmodified transcript of human mitochondrial tRNA^Asp^ shows an equilibrium between various secondary structures. It is suggested that the natural occurring modification m^1^A9, m^2^G10 and Ψ27 are important for correct folding. Queuosine at position 34 in the anticodon loop does likely not contribute to the secondary structure [55]. (**A**,**B**) adapted with permission from Motorin and Helm [40]. Copyright (2010) American Chemical Society; (**C**) The two methyl groups of m^2^_2_G at the exocyclic N2 of guanosine create a steric hindrance and preclude base pairing with cytosine. However, the methylations do not alter the base pairing with A or U as the amino group at N2 is not involved in this interplay. An interaction of m^2^_2_G with C is possible via a bridging water which leads to a greater distance between the two bases [56]; (**D**) Various examples show the impact of m^2^_2_G for a correct tRNA folding. In *Pyrococcus abyssi* tRNA^Pro^ contains m^2^_2_G at position 10, which wobble pairs with U25. A lack of this modification can lead to dramatic misfolding of the D-arm due to an extended D-stem [57]. tRNA^Asp^ of the same organism also contains m^2^_2_G10. However, this modification prevents a wrong base pairing with C27, which would lead to an enlarged D-loop [57]. In tRNA^Lys^ of *Haloferax volcanii*, m^2^_2_G can also direct the folding of the anticodon stem by interrupting C25-G45 and G26-C44 base pair which leads to an elongated anticodon and shortened D-stem [56]. A similar example is human cytosolic tRNA^Asn^. Here, m^2^_2_G is found at position 26 and prohibits base pairing with C11 [56].

For several non-modified mitochondrial tRNA transcripts, such misfoldings are described, and modifications are required to force the tRNA into its cloverleaf structure. A prominent example for the impact of such a simple base modification on a tRNA’s global structure is human mitochondrial tRNA^Lys^ (Figure 4A). The unmodified in vitro transcript forms an extended rod-like structure that has no similarity to the cloverleaf form of a tRNA. The introduction of a single methyl group at the adenine base at position 9 (m^1^A9), however, leads to the disruption of a Watson/Crick base pair with U64 and forces the folding into the canonical secondary structure [51,52]. This shows that one single methylation can lead to a dramatic change of the overall structure of the molecule. The structure-determining effect of the disruption of the A9-U64 base pair in the tRNA is further supported by the fact that replacing A9 by C or U64 by A or C, all disrupting the original base pair, lead to the formation of a functional cloverleaf structure. This indicates that the function of m^1^A9 is to avoid the base pairing that induces RNA misfolding [51].

Besides these massive structural rearrangements, m^1^A9 can also induce more subtle changes in a tRNA structure. In the nematode *Ascaris suum*, twenty of the twenty-two mitochondrial tRNAs lack the entire T-arm. All these twenty tRNAs carry the m^1^A modification at position 9, which is important for efficient aminoacylation and interaction with mitochondrial EF-Tu [26,53]. Structural investigations of mitochondrial tRNA^Met^ and tRNA^Phe^ show that this modification leads to a different folding pattern in the D-arm and the loop region that replaces the T-arm. These subtle rearrangements seem to affect the distance between CCA-end and anticodon, which in turn affects the binding efficiency of the corresponding aminoacyl tRNA synthetase or EF-Tu [26]. Hence, there is evidence that local, minor refoldings, which can affect the overall shape of the tRNA, represent an important prerequisite for tRNA functionality.

Another example where the introduction of modifications leads to a global structural rearrangement is the human mitochondrial tRNA^Asp^. For the in vitro transcript, an equilibrium of three different conformations was observed, where, besides the biologically active cloverleaf, two alternative structures exist: an extended hairpin and a cloverleaf-like form with bulged D-arm (Figure 4B) [23]. The mature in vivo tRNA carries four modifications, m^1^A9, m^2^G10, Ψ27, and Q34 and folds into the functional cloverleaf structure. As some of these modifications are only present partially, it is not yet certain which of them is responsible for the structural rearrangement. However, as Q34 is located in the anticodon loop, it can probably be excluded from contributing to the cloverleaf structure. Similarly, m^2^G10 is also unlikely to be involved, as the methylation does not alter the Watson/Crick base pairing properties of G10. Ψ27, though, is known to contribute the structural stability of bovine mitochondrial tRNA^Met^ [54]. Hence, it is possibly also involved in stabilizing the cloverleaf of the mitochondrial tRNA^Asp^. As shown in mt tRNA^Lys^, m^1^A9 has a changed Watson/Crick edge and cannot base pair with U, and it is very likely that the introduction of this single methyl group strongly contributes to the formation of the cloverleaf structure in the native tRNA. m^1^A9 is a characteristic modification of mitochondrial tRNAs and is rarely found in other tRNAs [18,23,27].

As mitochondrial tRNAs are A- and U-rich sequences, they can fold into many alternative—often nonfunctional—structures [18,58]. The introduction of this methyl group might represent a general strategy for such AU-rich tRNAs to avoid certain misfolded states, increasing the probability to adopt the canonical cloverleaf structure that represents a functional tRNA [51].

In thermophilic organisms, a bias towards GC-rich sequences is frequently observed, and the corresponding tRNAs are GC-rich as well [18,59,60,61,62]. This introduces a folding problem similar to the one observed for AU-rich sequences. G residues can also find many alternative base-pairing partners, which can lead to many different structures besides the cloverleaf. It is discussed that *N*^2^,*N*^2^-dimethylguanosine (m^2^_2_G) plays a similar structure-determining role in tRNAs of thermophilic archaea as m^1^A9 in mitochondrial tRNAs described above [57]. The m^2^_2_G modification eliminates a hydrogen-bond donor in the Watson/Crick edge, reducing the base-pairing properties of the modified G residue to G-U wobble and additional non-Watson/Crick base pairings like G-A interactions, while G-C pairing is no longer possible (Figure 4C). However, an alternative G-C base pair can be formed, where cytosine presents a different steric orientation and a water molecule is coordinated in the increased the space between the nucleobases (Figure 4C) [56]. In *Pyrococcus abyssi* tRNA^Pro^ contains m^2^_2_G at position 10 that is necessary to form a wobble interaction with U25. Without this modification the stem of the D-arm is extended leading to dramatic misfolding of the D-arm. In a second example, the m^2^_2_G modification at position 10 of tRNA^Asp^ prevents base pairing with C27, which would result in an enlarged D-loop (Figure 4C) [57]. In tRNA^Pro^ of *Haloferax volcanii*, m^2^_2_G at position 10 prevents the formation of G10-C23 or G10-C24 base pairs that would lead to a misfolded D-arm. Analogously, m^2^_2_G26 in tRNA^Lys^ of the same organism supports correct folding of the anticodon stem (Figure 4D) [56]. In thermophilic bacteria like *Aquifex aeolicus*, m^2^_2_G might also contribute to the stability of certain tRNAs. In tRNA^Cys^ of this organism, it is conceivable that the unpaired m^2^_2_G26 stabilizes the tRNA structure. Interestingly, the neighboring and originally base-paired G27 is also modified to m^2^_2_G. While at a first glance the resulting disruption of G27-C43 seems to destabilize the tRNA, the two methyl groups might form hydrophobic interactions with the neighboring G26 and C28 that lead to a greater contribution in structural stabilization than the hydrogen bonds of the G27-C43 base pair [63]. Interestingly, the prevention of tRNA misfolding by m^2^_2_G is not restricted to thermophiles, but is also observed in human cytosolic tRNA^Asn^, where m^2^_2_G is located at position 26, disrupting the formation of C11-G26 (Figure 4D). Instead, the modification results in a G26-A44 base pair at the top of the anticodon stem, forcing the transcript into the correct structure [56]. m^2^_2_G modifications with probably similar structural effects are described for tRNA^Tyr^ in several mammals [64,65]. These examples illustrate how *N*^2^,*N*^2^-dimethylguanosine represents a general tool to restrict the folding space of certain tRNAs, in which inactive, alternative structures are possible.

### 2.2. Local Structural Effects

**Figure 5:**
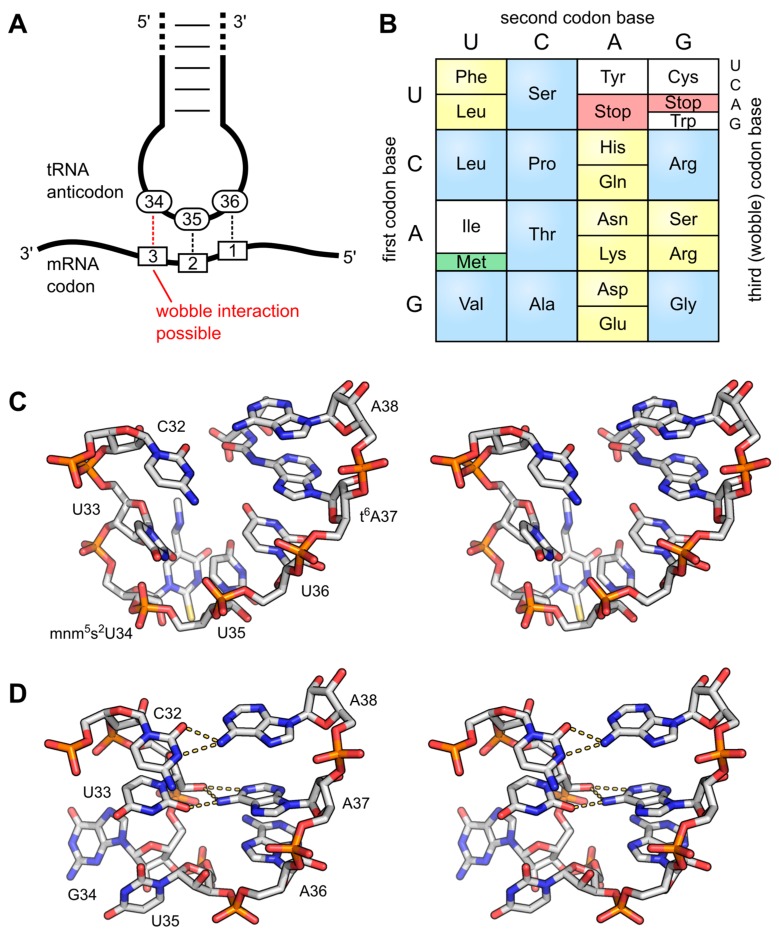
Modifications in decoding and anticodon loop structure. (**A**) The interaction of the anticodon bases (34–36) of a tRNA with the corresponding bases of the mRNA codons (3, 2, 1). A wobble interaction is possible between codon base 3 and anticodon base 34. The latter is frequently modified and directs the wobble interactions with the third codon base; (**B**) The standard genetic code is illustrated as a simple decoding table, 2-fold degenerate codon boxes are colored yellow, 4-fold degenerate boxes are blue. Start and stop codons are colored green and red, respectively; (**C**) Stereo image of the well-structured anticodon loop of tRNA^Lys^ from *E. coli*. Modifications mnm^5^s^2^U34 and t^6^A37 prevent wrong base pairing inside the 7-nucleotide loop and promote the formation of the conserved U-turn motif. The stacked anticodon bases are located on the same side of the loop. PDB: 1FL8 [91]; (**D**) Stereo image of a collapsed and unmodified anticodon loop of tRNA^Tyr^ from *Bacillus subtilis*. Here, bases 32 and 38 as well as 33 and 37 interact with each other and the U-turn motif is missing. The anticodon bases are not ordered and on opposite sides of the loop. PDB: 2LAC [89].

Besides such major rearrangements of the tRNA structure, modifications also trigger local changes in the molecule’s shape that are nevertheless equally important. In the anticodon loop, positions 34 and 37 are frequently modified and show a high abundance of complex hypermodifications. Their function has been investigated extensively and was greatly reviewed [34,66]. In principle, modifications in the anticodon loop fulfil two different functions: they modulate interaction possibilities between codon and anticodon and they fine-tune the tRNA structure. Specifically, base modifications at position 34, which is the first base of the anticodon, contribute to the wobble interaction with the third position of the corresponding codon in the mRNA (Figure 5A,B). A prominent example is tRNA^Ile^ carrying the anticodon UAU. In principle, this anticodon can read codons AUA (for isoleucine) and AUG (for methionine). Yet, it was shown in some instances that tRNA^Ile^ with unmodified UAU anticodon exists but has a strong preference for its cognate AUA codon, while it rarely misreads AUG [67,68]. In most organisms, however, tRNA^Ile^ carries the anticodon CUA. To avoid misreading of the methionine codon by this tRNA, C34 is modified to lysidine (k^2^C34, chemical structure shown in Figure 2), which restricts codon recognition to only AUA and thereby changes the amino acid identity of the tRNA from methionine to isoleucine [69,70]. In the archaeal species *Haloarcula marismortui*, *Methanococcus maripaludis* and *Sulfolobus solfataricus*, this tRNA^Ile^ carries a different modification at C34, fulfilling the same purpose of restricting the interaction to AUA codons. Here, the original cytosine is modified at the C2-oxo position, which is replaced by agmatine (decarboxy-arginine), resulting in agmatidine (C+ or agm^2^C) (see Figure 2) [71,72]. A complementary modification is that of *N*^4^-acetylcytosine (ac^4^C34, chemical structure shown in Figure 2) in elongator-tRNA^Met^ of *E. coli*, which prevents the recognition of the AUA isoleucine codon [73]. In non-plant mitochondria, however, both AUG and AUA codons are read as methionine [74]. Hence, mitochondrial tRNA^Met^ (carrying the anticodon CAU) has to recognize both codon forms. This is achieved by the introduction of 5-formylcytidine (f^5^C, see Figure 2) at position 34, a modification that pairs with both A and U residues at the corresponding codon position 3 [75].

In addition to these rather rare C-derived modifications, the majority of the manifold U34 modifications direct the wobble interactions of certain tRNAs by discriminating against closely related codons and simultaneously allowing the interaction with more than one codon for the same amino acid (2-fold and 4-fold degenerate boxes, Figure 5B). In bacteria and eukaryotes, xnm^5^U/xmo^5^U or xcm^5^U derivatives are found at this position, respectively. Interestingly, the wobble interaction between a U34 containing tRNA and a codon ending in G is weak. Therefore, in most cases, U34 has to be modified at position C5 of the uracil ring for efficient decoding of both purine-ending codons [76]. Additionally, the sulfur-containing 2-thiouridine (s^2^U34) and its derivatives preferentially base-pair with A at the third codon position, forming two hydrogen bonds. An interaction with G, which is usually possible for U, is less efficiently formed [77,78]. A study of different yeast strains cultured at elevated growth temperatures revealed divergent modification patterns at this tRNA position. Some typical laboratory strains lost the s^2^U34 modification at higher temperatures, whereas thiolation levels in closely related strains remained unchanged or even increased under the same conditions [79]. This example shows that one has to be careful with generalizations of conclusions drawn from the investigation of one model organism or strain. A different example of a modified uridine at position 34 is uridine 5-oxyacetic acid (cmo^5^U34) and additional 5-oxy derivatives (mo^5^U34, mcmo^5^U34) which can expand the repertoire of recognized nucleobases. Anticodons with such a modification can read not only codons ending with A or G, but also with U and sometimes even C, enabling the recognition of up to four codons, as in the case of tRNA^Pro^ in *Salmonella enterica* [80]. In certain cases, even an unmodified U34 is able to decode all four codons, as shown for *Mycoplasma capricolum*, where six of the eight four-fold degenerated codons are read by only one tRNA [81,82]. For a detailed description of the role of modified U34 in codon recognition, an excellent survey by Takai and Yokoyama is recommended [83].

The second function of modifications in the anticodon loop is a structural one. Here, modified bases reinforce a defined loop structure, a so-called U-turn. For efficient translation, the anticodon loops of all tRNAs have to adopt a highly similar conformation that promotes a stable codon-anticodon interaction in the ribosomal A-site. The canonical anticodon loop structure consists of seven unpaired nucleotide residues (Figure 5C). In a non-modified tRNA transcript, this loop is rather flexible and collapses into a structure with additional base pairs. In the in vitro transcribed *E. coli* tRNA^Phe^, this collapse results in the base pairs U32-A38 and U33-A37. As a consequence, the residual minimized anticodon loop consists of the three anticodon bases. This conformation is highly different from the canonical U-turn structure of a functional anticodon loop and thus results in reduced translation efficiency and fidelity [84,85,86]. In native tRNA molecules, this collapse is avoided by modifications of the highly conserved purine residue at position 37 (Figure 5C), which eliminates these unwanted base pairs and opens the loop into the correct structure [34,87,88]. Similar results were obtained for *Bacillus subtilis* tRNA^Tyr^ (Figure 5D). The introduction of i^6^A37 and Ψ39 led to the re-opening of the anticodon loop as investigated for tRNA^Phe^. However, the typical U-turn was not detected. The authors suggest that the missing queuosine at position 34 is responsible for its formation [89]. Purine 37 is frequently hypermodified, like wybutosine (yW), threonylcarbamoyladenosine (t^6^A) or 2-methylthio-*N*^6^-isopentenyladenosine (ms^2^i^6^A) and others [27,30,84,88]. Besides keeping the anticodon loop in a seven-nucleotide-organization, another function of the modified bases at position 37 (and also 34) is an improved stacking with neighboring residues due to an increased hydrophobic character of the modified bases [90]. These stabilizing interactions reduce the structural flexibility of the anticodon loop and reinforce the rather rigid and ordered U-turn shape of a functional anticodon conformation [34].

In addition to such a direct impact on the overall structure of a tRNA or its domains, modified nucleosides can also have indirect effects on the tRNA shape. It is well known that the compact L- form of a tRNA is dependent on the presence of bound Mg^2+^ ions that act as counter ions to shield the repelling negative charges of the phosphodiester backbone in interacting RNA domains [90,92]. Certain modifications can promote Mg^2+^ binding to other positions in the tRNA, resulting in strongly increased binding constants compared to the unmodified transcript [47,93]. A prominent example is 5-methylcytosine at position 40 (m^5^C40) in tRNA^Phe^ of *S. cerevisiae*. Residue 40 is located in the anticodon stem, two positions downstream of the anticodon loop. The Agris lab showed that this modification reinforces Mg^2+^ binding in the upper part of the anticodon loop, distant from m^5^C40 [94,95]. A similar observation was made for *E. coli* tRNA^Val^, where modifications were shown to strengthen the Mg^2+^ binding sites of the tRNA [93]. Furthermore, a strong metal binding site in cytosolic tRNAs was identified at position G15 in the D-loop [96], where a hydrated Mg^2+^ stabilizes a reverse Watson-Crick base pair G15-C48 (the Levitt base pair [97]) between D-loop and variable loop [98]. In archaea, a comparable stabilization of the Levitt base pair is achieved by a modification of G15, leading to archaeosine (G+; 7-formamidino-7-deazaguanosine, see Figure 2) [99,98]. While this archaea-specific modification interferes with Mg^2+^ binding, the positively charged formamidine group at the C7 atom fulfils the same function as the coordinated Mg^2+^ and stabilizes the tRNA structure in a similar way [98]. It seems that the combination of Mg^2+^ interaction and base modification contributes to the structural modulation of the tRNA and/or its individual domains [95].

tRNA modifications also affect the helix type that is formed as well as the overall rigidity and stability of the tRNA. While we have seen that methylations (m^1^A, m^2^_2_G) can influence the base pairing properties, they can also modulate the strength of individual hydrogen bonds in a base pair. One example is *N*^7^-methylguanosine (m^7^G), where the methyl group introduces a transient positive charge to the imidazole ring system, due to a temporary full protonation of the nucleoside [100]. This positive charge affects the non-Watson/Crick hydrogen bond between m^7^G at position 46 and G22 in the base triple C13—G22—m^7^G46 in yeast tRNA^Phe^. Similarly, m^1^A58, also fully protonated and positively charged, forms non-Watson/Crick hydrogen bonds with T54. As the purine ring of both methylated bases is located on the surface of the tRNA, it is discussed that these positively charged patches are involved in the specific recognition of the tRNA by proteins [100].

Furthermore, modifications can enhance the hydrophobic character of a base [90]. This leads to improved stacking in the helical environment, and, consequently, to the stabilization of the local structure in the tRNA. In a similar way, the enhanced stacking interactions of alkylated bases in the anticodon loop, even outside of the anticodon itself (position 37) contribute to codon-anticodon base pairing during translation [33,101,102]. Interestingly, pseudouridine Ψ, isosteric to its precursor uridine with an unchanged Watson/Crick edge, also has increased base stacking properties due to an elevated hydrophobic character [103]. A second way how Ψ can contribute to the stabilization of the tRNA structure is the formation of H-bonds with its additional H-bond donor N1 that is not present in U (see also the section on thermophilic adaptation, see Figure 3B for a three-dimensional structure of Ψ). However, when N1 is artificially methylated (m^1^Ψ) in order to inhibit H-bond formation in the anticodon loop of yeast tRNA^Phe^, the resulting base has the same stabilizing effect as the original Ψ—a clear indication that the improved stability results from the stacking properties of Ψ [104]. Yet, N1 as an H-bond donor represents an important feature of Ψ and contributes in several instances to the stability of the local tRNA structure. Representing one of the most abundant base modifications, the structure-stabilizing Ψ is found at a variety of different positions in tRNA transcripts [27,105]. In the TΨC-loop, where this name giving modification is particularly frequent, it forms a tertiary base pair with G18 in the D-loop [14]. In all three kingdoms, further pseudouridine positions are distributed over the whole tRNA structure, where they might also contribute to the stabilization of the correct shape [18,105,106,107,108]. A very interesting and highly abundant Ψ site is position 13, located at the distal end of the D-stem. Together with position 22, position 13 forms the terminal base pair of this stem. Here, Ψ seems to fulfil two functions. As a general effect of this rigid modification, the local structure of this tRNA domain is stabilized [109,110]. Furthermore, Ψ forms base pairs with all four canonical A, U, G and C bases that are more stable than those with the unmodified U [111,112,113]. Especially when U22 or G22 is present, Ψ13 is found in 96% or 75% of analyzed tRNA sequences, respectively [109]. With A22, however, no such preference for Ψ13 is observed, as an unmodified U13 obviously forms a base pair with sufficient stability. These facts indicate that the main function of Ψ13 is probably to stabilize the end of the D-stem, regardless the nature of the interacting position 22.

For several of these positions, molecular dynamics (MD) simulations of the yeast tRNA^Asp^ anticodon arm and structural analyses of *E. coli* tRNA^Gln^ have shown that N1 interacts with the neighboring upstream nucleotide by hydrogen bonding with a bridging water molecule that forms additional H-bonds to the adjacent phosphate groups [108,114]. This link between the tRNA backbone and Ψ stiffens this region and increases the rigidity of the local tRNA structure. Recent thermodynamic analyses of RNA duplexes with defined Ψ positions confirmed an enhanced structural stability compared to hairpins lacking this modification [112,113]. In addition, pseudouridine exhibits a third stabilizing effect that is mediated by its propensity to keep the ribose moiety in a C3’-endo conformation (Figure 3B) [103,115,116]. Accordingly, Ψ reinforces the more stable A-helical form in the tRNA domains.

A similar structural reinforcement that affects the sugar pucker in tRNA is mediated by modifying the ribose itself. The introduction of a methyl group at the 2′OH of the ribose promotes a C3′-endo conformation, because in a C2′-endo pucker, the large methyl group leads to a steric clash with the 3′-phosphate and the base, especially pyrimidines [115,117]. Hence, 2′O-methylated residues adopt a C3′-endo conformation and, consequently, an A-helix is preferred. In the case of Gm18 (found in eukaryotes and bacteria), for instance, the C3′-endo-mediated local rigidity may affect the stability of the D-arm [117,118]. Furthermore, as Gm18 interacts with Ψ55, it also stabilizes the L-shape of the tRNA [14]. In archaea, C56, located in the TΨC loop, is methylated to Cm56 [119]. This position interacts with G19 in the D-loop, and the rigid Cm56 might also contribute to the stability of these tertiary interactions in the tRNA’s 3D structure. Such ribose methylations are not restricted to tRNAs, but are found in many different types of RNA, predominantly in thermophilic organisms [120,121]. In general, all modifications that stabilize the tRNA structure also represent an adjustment to thermal changes as outlined in more detail in the next section. Besides such structural effects, 2′O-methylations contribute to a general stability of (t)RNA against hydrolytic damage, as an in-line nucleophilic attack of the 2′OH group [122], leading to backbone breakage, is no longer possible. In tRNA, this ribose modification is frequently found at positions 4, 6, 18, 32, 34, 39, 44, 54 and 56 [117,123,124]. Interestingly, positions 32, 34 and 54 can also carry 2-thiolated uridines or cytosines [117]. In the archaeon *Pyrobaculum aerophilum*, position U8 is also predicted to be modified to Um8 [125,126]. As U8 also represent a conserved site for thiolation to s^4^U as a UV protective [127], it is possible that in this case, position 8 is also further modified to s^4^Um8. While thiolation can change the base pairing properties, as described above for 2-thiouridine (s^2^U34), the second effect of 2-thiolation is a conformational one, comparable to the ribose methylation. The steric clash of the bulky 2-thiocarbonyl group with the 2′OH of the ribose stabilizes the C3′-endo form of the sugar pucker [128,129]. Hence, this ribose conformation can be promoted by modifications of both parts, base or sugar, that lead to a steric hindrance in a C2′-endo conformation.

However, not all modifications stabilize a tRNA structure and increase its rigidity. A whole plethora of experiments show that tRNAs have to exhibit a certain flexibility for proper function in maturation, aminoacylation, translation as well as regulation of gene expression [130,131,132,133,134,135,136,137]. While the stability and (non-)isostericity of individual base pairs (e.g., GC versus GU) can increase the local flexibility in a tRNA domain [137], there are also modifications that contribute to the conformational elasticity of these molecules. Dihydrouridine (D, see Figure 3C for a three dimensional structure) is the name-giving modification that is frequently found in the D-loop, predominantly at positions 16, 17, 20, 20a and 20b, and additionally at position 47 in the variable loop [18,27,99,138,139,140,141,142,143]. It is formed by a reduction of the double bond between positions C5 and C6 in the pyrimidine ring of uridine [144]. Due to the saturation of the C5–C6 bond, D is the only described non-aromatic base found in nucleic acids [90]. The base is no longer planar, but puckered, and positions C5 and C6 are displaced on opposite sides of the plane consisting of positions N1, C2, N3 and C4, resulting in a half-chair conformation [145,146]. The saturated non-planar shape of D has two structural consequences. First, D is unable to stack with neighboring aromatic bases. Second, at position C6, D carries a methylene group with a larger volume compared to the corresponding position in uracil. Due to this methylene group, D is sterically restricted and prefers a C2′-endo conformation of its ribose [147,148]. This sugar pucker is also relayed to the neighboring base located upstream, increasing the local structural rearrangement induced by this modification [149]. Hence, the combined influence of dihydrouridine (destacking of bases, preference of C2′-endo conformation, induction of C2′-endo pucker in the 5′-neighboring nucleotide) introduces local and functionally important flexibility in the tRNA molecule. Nevertheless, D can also induce stability in a neighboring helical region, as shown in the D-arm of *Schizosaccharomyces pombe* tRNA_i_^Met^, where D enhances the flexibility of the D-loop, while it simultaneously forces the D-stem to adopt a rather stable conformation [41]. Yet, it came as a surprise that the lack of D20a in the *E. coli* tRNA^Ser^ lowered the melting temperature, indicating that the presence of this modification contributes to tRNA stability [99]. It is discussed that the D-mediated locally increased flexibility may facilitate the formation of the stabilizing neighboring tertiary base interactions in the elbow region of the tRNA. In the same series of experiments, it was shown that these tertiary interactions are then further stabilized by the modification of the involved bases like the above mentioned s^4^U8 that pairs with A14 in the D-loop (Figure 1) [99].

Taken together, post-transcriptional modifications are essential in modulating the structure of a tRNA. Besides large rearrangements that force the transcript into its canonical cloverleaf shape, modifications are involved in structural fine-tuning of different positions of individual tRNAs. In addition, the rigidity and flexibility of the whole transcript or individual domains is regulated by modified nucleosides. As the structural rigidity of a biomolecule is an important hallmark in thermal adaptation, these modifications contribute to tRNA functionality in thermo- and psychrophilic organisms. In the following section, such thermal adaptations due to tRNA modifications will be discussed.

## 3. Modifications and Temperature Adaptation

In the biological habitats on Earth, the environmental temperature ranges from below 5 °C to above 100 °C. While the moderate habitats are populated by mesophilic organisms, we find specifically adapted species thriving in cold and hot environments. As temperature is an important parameter in terms of reactivity and stability, many adaptations of biomolecules in psychro- and thermophilic organisms are known. Interestingly, many of these strategies for cold or heat adaptation are not specific for a certain type of biomolecule (e.g., proteins), but can be found in other cellular components, like (t)RNA as well.

### 3.1. Thermophilic Adaptation

To render proteins functional at high temperatures, a series of adaptations, individual or in combination, are possible. In general, the rigidity of the molecule is increased in order to prevent thermal denaturation. Here, an increased number of salt bridges, disulfide bridges and hydrophobic interactions is frequently observed. In addition, chaperones help proteins to keep their active functional conformation. Finally, glycosylation can help to stabilize proteins at elevated temperatures [150]. In principle, very similar strategies are employed by thermophilic organisms to increase the stability of their nucleic acids (reviewed in [151]).

tRNA molecules—and other nucleic acids—exposed to heat are confronted with two phenomena that affect their functionality: The first is thermal denaturation, where the essential 3D and 2D structure is destroyed, as the transcript melts down into its unfolded—and nonfunctional—state. The second phenomenon is thermal degradation, leading to irreversible Mg^2+^-induced hydrolytic cleavage of phosphodiester bonds [152]. Other damages like depurination at dihydrouridine positions can occur as well [153]. Hence, organisms thriving at high temperatures have an urgent need for thermal stabilization of their tRNAs. One predominant temperature-dependent adaptation is the increase of the GC-content especially in the stems of the cloverleaf structure [60,61]. Oshima reported that an increase of 5% in the GC-content raises the melting temperature of a tRNA by 1.5 °C [62]. However, as mentioned above, a high GC-content is bought at the expense of increased possibilities for misfolding of the tRNA.

A high level of GC alone does not explain the observed thermal stability of tRNA. In the hyperthermophile *Pyrococcus furiosus*, it was shown that tRNA has a melting temperature that was about 20°C higher than what was calculated solely from the GC-content. This indicated that additional stabilizing factors must be involved [121], which render these tRNAs thermostable. Besides modifications, polyamines and RNA chaperones have been found to be important for tRNA stabilization. Many thermophilic organisms produce long and branched, positively charged polyamines like 4,4-bis(3-aminopropyl)-1,8-diamino-4-azaoctane, 4-(3-aminopropyl)-1,7-diamino-4-azaheptane or *N*^4^-bis(aminopropyl)spermidine that interact with negatively charged nucleic acids and also stabilize tRNA structures [154,155,156,157]. Furthermore, the interaction with proteins also contributes to tRNA stability. In *Aquifex aeolicus*, a small protein was identified that seems to stabilize the tRNA’s L-shape at high temperatures [158], and even tRNA modification enzymes per se help the tRNA to adopt and maintain its functional structure [159,160].

A third strategy to protect tRNAs from denaturation and degradation is the introduction of certain modifications. Accordingly, tRNAs from thermophiles show a larger abundance and diversity of modifications than their mesophilic or psychrophilic counterparts [121,161,162,163]. As indicated above, thiolations, methylations and even double methylations introduce structural rigidity and, consequently, thermal stability of tRNAs. 2′O-methylations are highly effective in blocking temperature-induced hydrolytic damage of the tRNA backbone, as a nucleophilic attack of the 2′OH on the neighboring phosphodiester bond is prevented. Especially in archaea, this 2′O-methylation is frequently found in combination with a methylated base, resulting in doubly modified nucleosides like m^2^Gm, m^2^_2_Gm, ac^4^Cm and others [121,164,165,166]. Besides increasing the local rigidity, ac^4^Cm promotes the C3′-endo conformation of the adjacent ribose [166]. Hence, the corresponding position in the tRNA is not only protected from hydrolysis, but contributes to the formation of a stable A-helix. As mentioned above, pseudouridine (Ψ) stabilizes the tRNA structure by improved stacking properties, the presence of an additional H-bond donor and the preference of the C3′-endo sugar pucker. The latter is also passed on the neighboring nucleotides, which then favor this stable ribose conformation as well [103]. Consequently, Ψ is a tRNA modification that contributes to higher thermostability as shown for the anticodon stem-loop of tRNA^Lys^. In this example, the modification of U39 to Ψ39 leads to a 5 °C higher melting temperature due to improved base stacking and a strengthening of the base pair with A31 that closes the helix [167]. A TruB defective *E. coli* strain, which lacks the well conserved Ψ55 in the T-loop, showed less tolerance to thermal stress, indicating that this modification is involved in tRNA stability at high temperatures [168].

To keep the tRNA in its L-shape, important thermo-stabilizing modifications are found in the elbow region of the tRNA, where tertiary interactions between D- and T-loop are essential. As mentioned previously, U54 is usually fully methylated at position C5, resulting in T54 (ribothymidine or m^5^U). In many thermophiles, this base is further modified by the introduction of a thiocarbonyl group at position 2, leading to 2-thioribothymidine (s^2^T54). This modification increases the melting temperature of a tRNA by 3 °C [169,170] by promoting C3′-endo sugar puckering as well as a tertiary interaction with A58, where a reverse-Hoogsteen base-pairing occurs. Interestingly, in most archaea, T54 is replaced by Ψ54 or m^1^Ψ54 [171,172]. As these modifications have base pairing properties and structural shapes highly similar to uracil or ribothymidine [173], it is likely that they fulfil a similar structural function in stabilizing the shape of the TΨC loop and its tertiary interaction with the D-loop. As T54, Ψ or m^1^Ψ can form the described reverse Hoogsteen pair with A58 [174,175]. Furthermore, this base pair is sandwiched between the neighboring base pairs Ψ55-G18 and G53-C61, and it is very likely that Ψ54 or m^1^Ψ54 further increase the thermal stability of the elbow region due to their enhanced stacking properties described above [174]. The interaction partner A58 is often modified to *N*^1^-methyladenosine (m^1^A58), especially in thermophiles. While this modification per se does not contribute to thermostability, it is a prerequisite for the introduction of s^2^T54, as it represents an essential recognition element for the modifying enzyme s^2^T-thiolase [176]. The dependence of one modification on another adds an additional layer of complexity to the identification of modified, thermostabilizing nucleosides. When the methylation of A58 is blocked in a corresponding knock-out strain of *Thermus thermophilus*, the strain becomes thermosensitive, because s^2^T54 cannot be formed [177]. A second example in *T. thermophilus* is *N*^7^-methylguanine at position 46 (m^7^G46). A knock-out strain lacking this modification has severe growth defects at 80 °C, and several tRNAs had a decreased melting temperature and half-life, due to hypomodification at several positions [178]. This indicates m^7^G46 is a prerequisite for efficient modification of further positions that are then required for stabilization. In addition, recent experiments indicate that polyamines not only stabilize the tRNA structure as described, but are also important for the introduction of modifications, as they can enhance the activity of the corresponding enzymes [179]. Hence, the distinct identification of an individual modification that contributes to thermal stability is not an easy task, as additional features like modification networks, substrate recognition, and enhancement of enzyme activity have to be considered.

### 3.2. Psychrophilic Adaptation

Besides high temperature environments, cold habitats are equally challenging for biological systems, as low temperatures negatively influence membrane fluidity, water viscosity, solute diffusion rates, enzyme kinetics and macromolecular interactions [180,181]. Hence, to remain functional, adaptations of biomolecules are likewise required. As the majority of biological habitats are temporarily or permanently exposed to temperatures below 5 °C, the need for psychrophilic adaptation should not be underestimated [180,182,183]. For cold-adapted proteins, an increased structural flexibility seems to represent a major way to render these macromolecules functional [184,185,186]. Yet, such an increase in flexibility can be difficult to determine, as it can be restricted to local regions of the macromolecule, for instance the catalytic core of an enzyme [185].

The adaptation of RNA to low temperatures is still poorly understood. In contrast to mesophilic or thermophilic organisms, the genome of psychrophilic organisms carries an increased amount of tRNA genes [180]. This could be a possible compensation of the decelerated transcription rate due to low temperature [60]. Furthermore, one might expect that psychrophilic tRNAs have a decreased amount of GC-residues to increase flexibility. Indeed, in the antarctic shrimp *Euphausia sperba*, a loss of contiguous G-C pairs and a replacement by G-U in the T-arm were reported, resulting in a reduced thermal stability and cold adaptation [187]. However, in several other psychrophiles, a GC-content of 54%–59% is described, which is comparable to that of mesophilic tRNAs [60,161]. While proteins consist of 20 different amino acids, (t)RNA contains only four building blocks, reducing the potential for adaptation by sequence diversity. Hence, post-transcriptional modification plays an important role. As discussed above, most modifications lead to an increased molecular stability, while only a small minority increases the flexibility of a tRNA. Here, the non-aromatic dihydrouridine is of particular importance. It is the only tRNA modification that is unable to stack with other bases and supports the C2′-endo sugar pucker. As a consequence, dihydrouridine-containing regions are more flexible and have a wider range of glycosyl-torsion angles [149,188]. Dalluge et al. could show that the equilibrium between C2′ and C3′-endo conformation of dihydrouridine and the 5′-adjacent ribose is strongly temperature dependent and shifts towards C2′-endo at lower temperatures [149]. Consequently, the tRNA retains its local flexibility in the cold. Such a locally restricted flexibility was demonstrated by melting experiments, where psychrophilic tRNAs revealed a melting temperature similar to that of mesophilic tRNAs [161]. Hence, similar to proteins, an increased local flexibility in tRNAs represents an important adaptation to a cold environment. Many psychrophilic organisms show an increased dihydrouridine content in tRNAs, while mesophilic and thermophilic organisms have only very low amounts of this modification in their tRNAs to adapt to high temperatures [120,161,163].

As many microorganisms have to deal with a broad temperature range in their habitats, it is often difficult to identify simple cold or heat adapted tRNAs. In a knock-out strain of the thermophile *T. thermophilus*, the lack of a stabilizing Ψ55 modification leads to a growth retardation at lower temperatures (50 °C), while growth at high temperatures is not affected [189]. Obviously, this strain has lost its low-temperature adaptation. Nucleoside analysis of the corresponding tRNAs revealed an abnormally high content of m^1^A, Gm, m^1^G and s^2^T in the mutant. Furthermore, tRNAs from the mutant strain isolated at 50 °C showed an 8 °C higher melting temperature compared to wild type tRNAs. At 70 °C, however, mutant and wild type tRNAs had similar melting temperatures. These results indicate that nucleoside modifications are introduced in a network-like manner, where Ψ55 modification represents a control mechanism for thermal adaptation of tRNA functionality. For adaptation to low temperatures, the presence of Ψ55 restricts the incorporation of modifications conferring thermostability, while its absence enhances the addition of such modifications, increasing thermal stability. Further, a recent report indicates that the neighboring modification m^5^U54 supports Ψ55 in preventing excess of the thermostabilizing modified nucleosides, adding another layer of complexity to this modification network [190]. Hence, as a modification originally identified to stabilize a tRNA structure, Ψ55 can also contribute to its flexibility and functionality at low temperatures. The fact that organisms can modulate nature and amount of tRNA modifications as an adaptation to changing environmental temperatures demonstrates the complexity of modification networks and difficult interpretation of modification patterns.

## 4. Regulation of Modifications

While the number of modified nucleosides in tRNA and other RNA species is steadily increasing, the introduction of such post-transcriptional modifications, a very complex enzymatic process, is still not fully understood. MODOMICS, a database for RNA modification pathways, currently contains 144 different RNA modifications and collects data on their synthesis [106]. About 1% of the genes in *E. coli* code for modification enzymes and similar estimates were made for yeast. In *Mycoplasma capricolum* even 4% of the genome correspond to modification enzymes, reflecting the significance of post-transcriptional modifications [191,192]. Recently, efforts were made to predict the modification pattern of tRNAs based on the presence of genes coding for modification enzymes in the organism’s genome [193]. Some enzymes like yeast pseudouridine synthases 3 and 4 (Pus3 and Pus4) are region-specific and introduce a Ψ at position 38/39 and 55 of tRNAs, respectively [194,195]. In contrast, Pus1 is a multi-specific enzyme and pseudouridinylates various positions in the anticodon arm and T-arm [196]. Such substrate variety impedes a precise prediction of modification sites and, moreover, some positions are not necessarily modified although the corresponding enzymes are genomically encoded. As already discussed, some modifications can represent a prerequisite for the introduction of further modifications, but these interplays are also not sufficiently understood, which hampers a prediction even further. Early experiments revealed that some modifications are introduced at the level of primary transcript, whereas others are incorporated into the mature tRNA. This reflects the different capabilities of modification enzymes to recognize tRNAs. Indeed, in 1996 Grosjean et al. established the classification of tRNA-modifying enzymes in two groups according to their sensitivities to structural perturbations. Enzymes which only need a local structural element, like the acceptor domain, for productive recognition are assigned to group I. The interruption of tertiary interactions can favor an efficient reaction for these enzymes [197,198]. Group II includes enzymes that are sensitive to perturbations in the tRNA structure. This group is further separated into enzymes requiring an intact L-shaped tRNA (group IIa) and enzymes tolerating certain structural deviations (group IIb) [199]. Using microinjection of yeast tRNA^Tyr^ precursors into *Xenopus* oocytes, it was revealed that the modifications m^5^C49, T54, Ψ55 and m^1^A58 were incorporated to substrates containing intron and 5′-leader sequence before any modification in the D-arm occurred [200]. The anticodon loop was modified only after intron removal. These findings suggest that there might be a general order of modification events. First, modifications occur in the sufficiently structured T-arm which then promotes proper folding of the D-arm and enables subsequent modification.

The chemical modification of tRNAs has been proven to be quite dynamic, and not as static as has been historically assumed. Instead, the dynamic addition or removal of modifications has been increasingly suggested to serve as signal for the regulation of biological processes [201]. For example, highly sensitive mass spectrometry measurements revealed that a set of tRNA modifications changed upon exposure to different toxins [202], and under oxidative stress or in response to growth arrest conditions, such as nutrient depletion, 5-methylcytosine levels were shown to increase in certain yeast tRNAs [203,204]. This illustrates how the investigation of modification patterns and dynamics opens up a new frontier in tRNA research with an additional level of complexity.

Additionally, modifications have been described in recently found non-coding RNAs termed tRNA-derived fragments (tRFs) which represent tRNA cleavage products formed under stress conditions [205]. These fragments are thought to play a role in regulation of various cellular functions and also in cancer [206]. Evidence suggests that tRNA modifications can regulate the formation of tRFs. For example, m^5^C38 in tRNA^Asp(GTC)^, tRNA^Gly(GCC)^ and tRNA^Val(AAC)^ reduces cleavage during heat shock response in *Drosophila* [207,208].

Lastly, orthogonal tRNAs, heavily used in the field of genetic recoding, have recently been shown to serve as targets for modification enzymes [209]. These tRNAs in combination with an orthogonal aminoacyl tRNA synthetase are introduced into organisms to expand their genetic code so that additional, non-canonical amino acids (ncAA) can be incorporated into cellular proteins. By including an ncAA, the repertoire of chemical functionalities is expanded and thus the activities and properties of proteins synthesized by the cell’s translation machinery can be altered. It was shown that the orthogonal tRNA^optAUG^ is a substrate for TadA, an A to I editing deaminase in *E. coli* that is usually involved in essential maturation steps of endogenous tRNAs [209]. Reverse transcription, amplification and sequencing experiments demonstrated that the adenosine at position 34 of the orthogonal tRNA was modified to inosine. As a result, the codon recognition properties of the tRNA were drastically altered, and the tRNA^optAUG^ failed to discriminate between the two histidine codons CAU and CAC.

These examples show that post-transcriptional modifications are ubiquitous and found in all tRNA transcripts—natural ones, orthogonal transcripts, and tRNA fragments thereof. Moreover, it is evident that modifications are closely tied to tRNA functions and can enable precise fine-tuning and necessary adaptations to environmental stresses. In some cases, tRNA modifications and their functions are well known, but for the majority of instances their impact on biology has to be investigated in more detail.

## 5. Conclusions

Although RNA only consists of four elemental units, its structures and functions are manifold and further improved and expanded by the introduction of post-transcriptional modifications. Such nucleoside alterations are not restricted to tRNA, but found in many different transcripts, where they affect cellular processes like splicing, translation and RNA degradation. Continuous progress in sequencing technology will allow us to advance the investigation of modifications, resulting in the detection of further modifications in all kinds of transcripts. In case of tRNA, modifications ensure correct folding, efficient translation, and can serve as identity elements for recognition by aminoacyl-tRNA synthetases. Moreover, modifications can adapt tRNAs to different environmental conditions such as temperature. Furthermore, modified bases can also be of great importance for applications in synthetic biology to fine-tune the structure of aptamers and riboswitches or for the improvement of orthogonal suppressor tRNA systems in the introduction of non-natural amino acids into proteins. While our knowledge about the precise function of most modifications improved dramatically over the last decades, further research is required to understand the structural and regulatory impact of these fascinating nucleoside varieties.
